# Supplementary material for: Proteomic landscape of Ewing sarcoma primary tumors and metastases
Source: Nat Commun. 2026 Mar 11;17:3802. doi: 10.1038/s41467-026-70449-5 (PMC13111610; doi:10.1038/s41467-026-70449-5)
Supplement: Supplementary file 1 — Supplementary Information [file 41467_2026_70449_MOESM1_ESM.pdf]

Gordon et al. Supplementary Figure 1

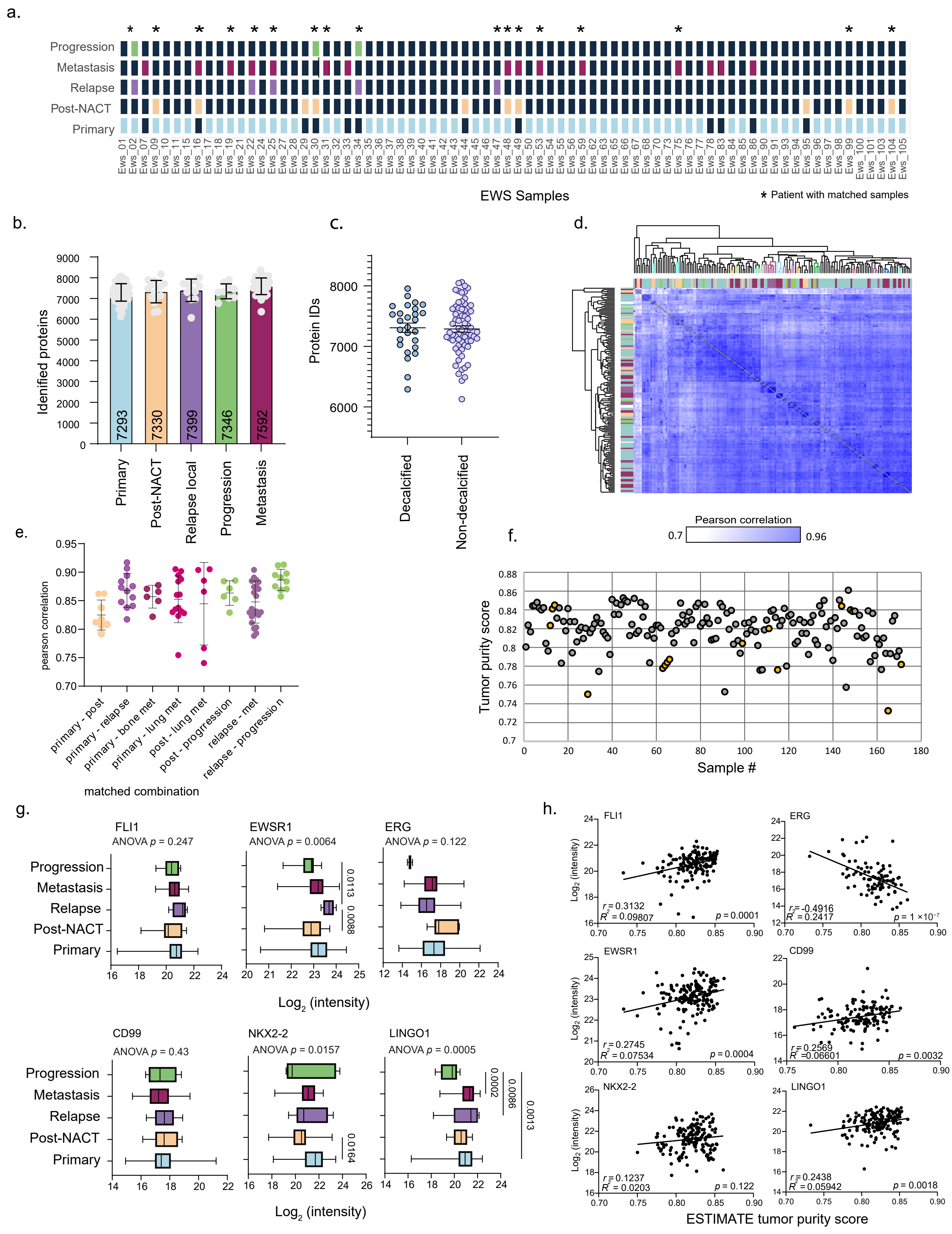

Supplementary Figure 1: **Proteomics of EWS cohort**

a) Illustration of the matched nature of the EWS cohort (*n* = 17 biologically independent patients). Seventeen patients had samples from multiple disease stages. Colored rectangles (non-black) indicate analyzed samples; samples are color-coded by tumor stage (as defined in Figure 1e).

b) Bar plot of the average number of quantified proteins in each sample type (*n*= 92 primary, *n* = 15 post-NACT, *n* = 12 relapse, *n* = 9 progression, *n*= 34 metastasis samples). Data are presented as mean ± SD. Each dot represents a sample.

c) Number of identified proteins in decalcified (*n* = 27) and non-decalcified (*n* = 65) primary EWS tumor samples. Data are presented as mean ± SEM.

d) Pearson correlation matrix of all samples (*n* = 170 biologically independent samples). Each row and column represents an individual sample. Samples are color-coded by tumor stage. Pearson correlation coefficients range from ***r*** = 0.70 to 0.96. Colored sample pairs that are co-clustered show samples from the same patients.

e) Average pairwise Pearson correlations between cancer progression stages. Data are presented as mean ± SD.

f) Dot plot shows the tumor purity score for each sample as determined by the ESTIMATE algorithm based on the proteomics data. The x-axis represents the sample ID and the y-axis represents the Tumor Purity Score, ranging from 0 to 1, where 1 indicates the highest purity. Post-NACT samples are color coded in orange.

g) Box plots show expression ranges of six EWS-associated proteins across tumor stages (*n* = 88-162 biologically independent samples). The center line represents the median, box limits represent the upper and lower quartiles, and whiskers represent the minima and maxima. Statistical significance was determined by one-way ANOVA followed by two-sided Tukey's multiple comparison test. P-values are indicated in the graph where significant

h) Pearson correlation of EWS-associated proteins (log_2 intensity) with the ESTIMATE tumor purity score (*n* = 88-162 biologically independent samples).

Source data are provided as a Source Data file.

Gordon et al. Supplementary Figure 2

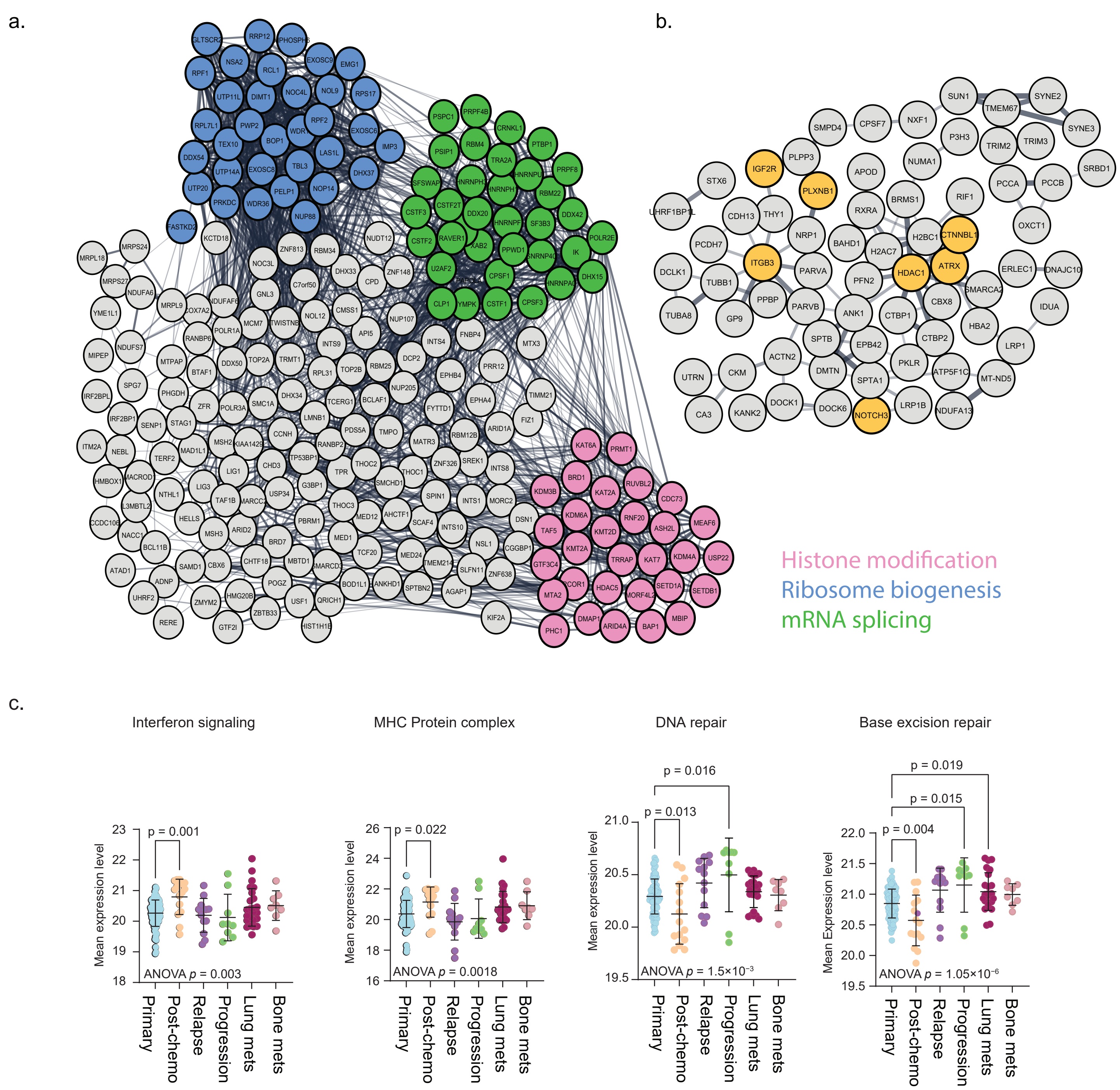

Supplementary Figure 2:

a) A protein network of cluster 4, consisting of proteins downregulated upon chemotherapy. Protein-protein interactions were taken from the STRING database, and the network visualization was edited in Cytoscape. Key functional clusters are highlighted, including ribosome biogenesis (blue), mRNA splicing (green), and histone modification (pink).

b) A protein network of cluster 1, consisting of proteins downregulated relative to the primary tumors. Protein-protein interactions were taken from the STRING database and the network visualization was edited in Cytoscape. Proteins known to mediate EWS-ETS signaling are specifically highlighted (orange).

c) Dot plots of selected enriched pathways. Each dot represents a patient, and the y-axis shows the mean expression level of all proteins in this pathway per patient. Statistical analysis was performed using one-way ANOVA to assess overall differences, followed by two-sided Dunnett's multiple comparisons test between primary and other tumor stages (n = 6 groups; total n = 162 biologically independent samples). Data are presented as the mean ± SD. P-values are indicated within the figure panels.
Source data are provided as a Source Data file

Gordon et al. Supplementary Figure 3

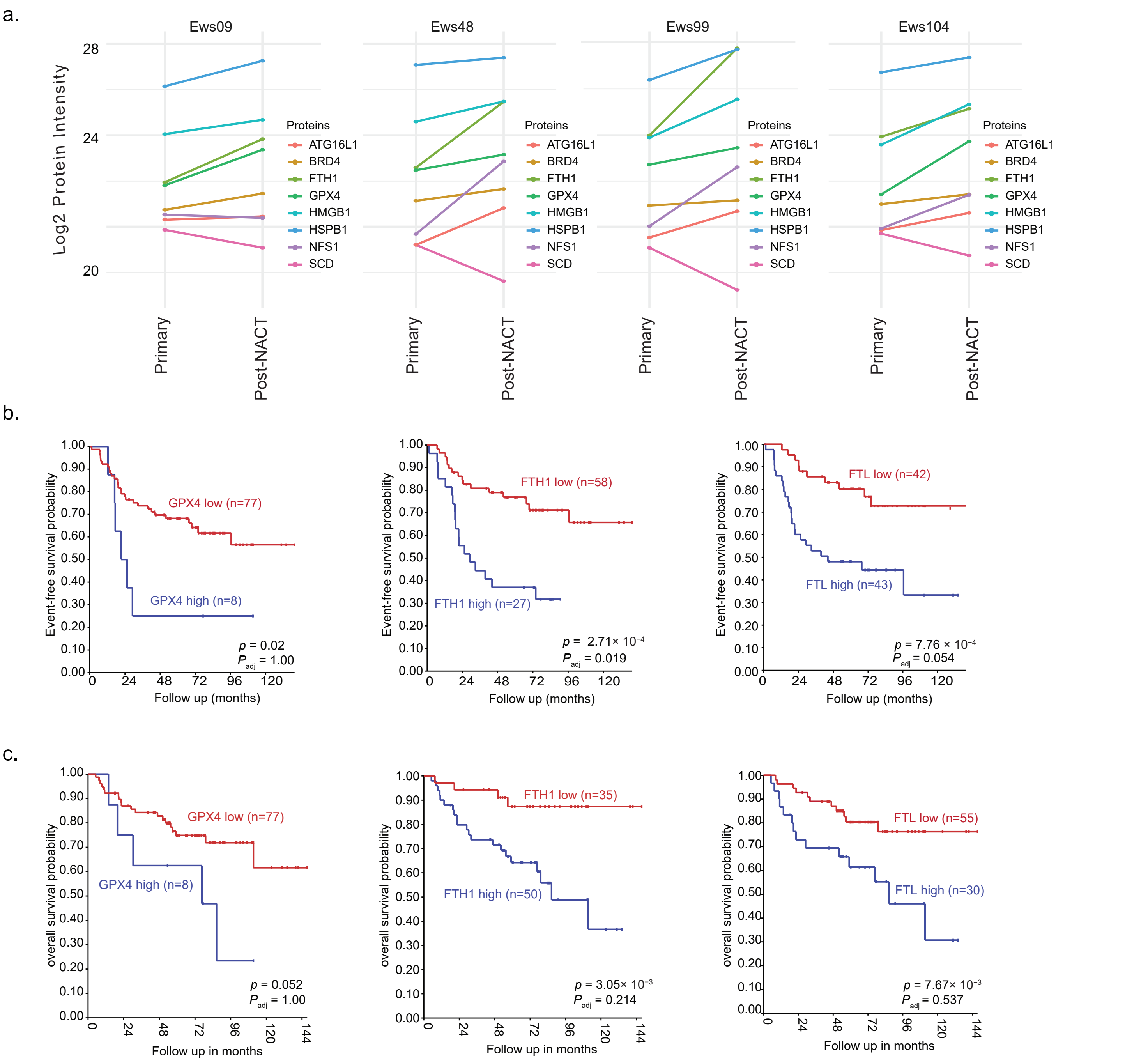

Supplementary Figure 3:

a) Ferroptosis-related protein changes (log2 intensity) in matched primary tumors and post-NACT samples (paired two-sided Student's T-test, $p < 0.05$). Data is shown for n = 4 patients (one panel per patient); each line represents a specific protein.

b,c) Kaplan Meier survival analysis of a publicly available mRNA expression analysis of EWS (Dirksen dataset, n = 85) shows shorter event-free survival and overall survival for tumors with high GPX4, FTH1, and FTL expression. Statistical significance was determined using the two-sided log-rank test (P-values are indicated in the figure). Additional survival analysis using univariable Cox proportional hazards regression (for continuous variables) was performed; full statistical details, including Hazard Ratios, 95% confidence intervals, and Wald test statistics, are provided in the Source Data file. Source data are provided as a Source Data file.

Gordon et al. Supplementary Figure 4

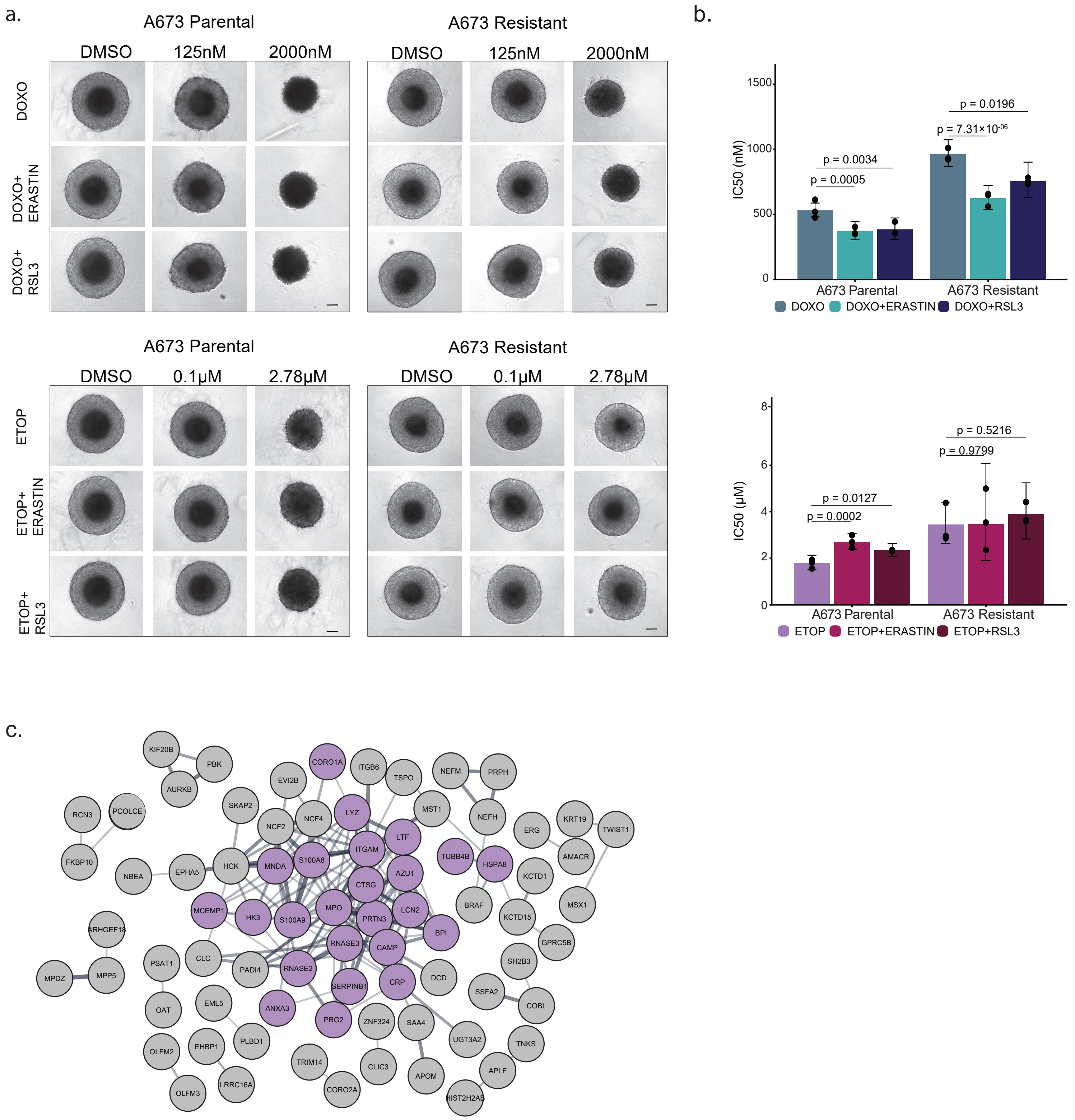

Supplementary Figure 4:

a) Representative images of A673 parental and resistant spheroids treated with chemotherapy alone or in combination with RSL3 or erastin. Images are representative of n = 3 independent spheroid replicates. Scale bar, 200 μm.

b) Bar plots show chemotherapy IC_{50} values in A673 spheroids treated with doxorubicin or etoposide alone, or in combination with RSL3 or erastin (fixed concentrations: 30nM or 0.4 μM, respectively). Data represent the best-fit IC_{50} values from n = 3 independent spheroid replicates. Error bars indicate the 95% confidence interval of the fit. Statistical significance was determined using the extra sum-of-squares F-test. P-values are shown in the figure.

c) A network of proteins that were significantly higher in primary tumors with relapse within the non-metastatic group. This network was generated using the STRING database and visualized in Cytoscape. Proteins related to neutrophil degranulation are colored purple.

Source data are provided as a Source Data file.

Gordon et al. Supplementary Figure 5

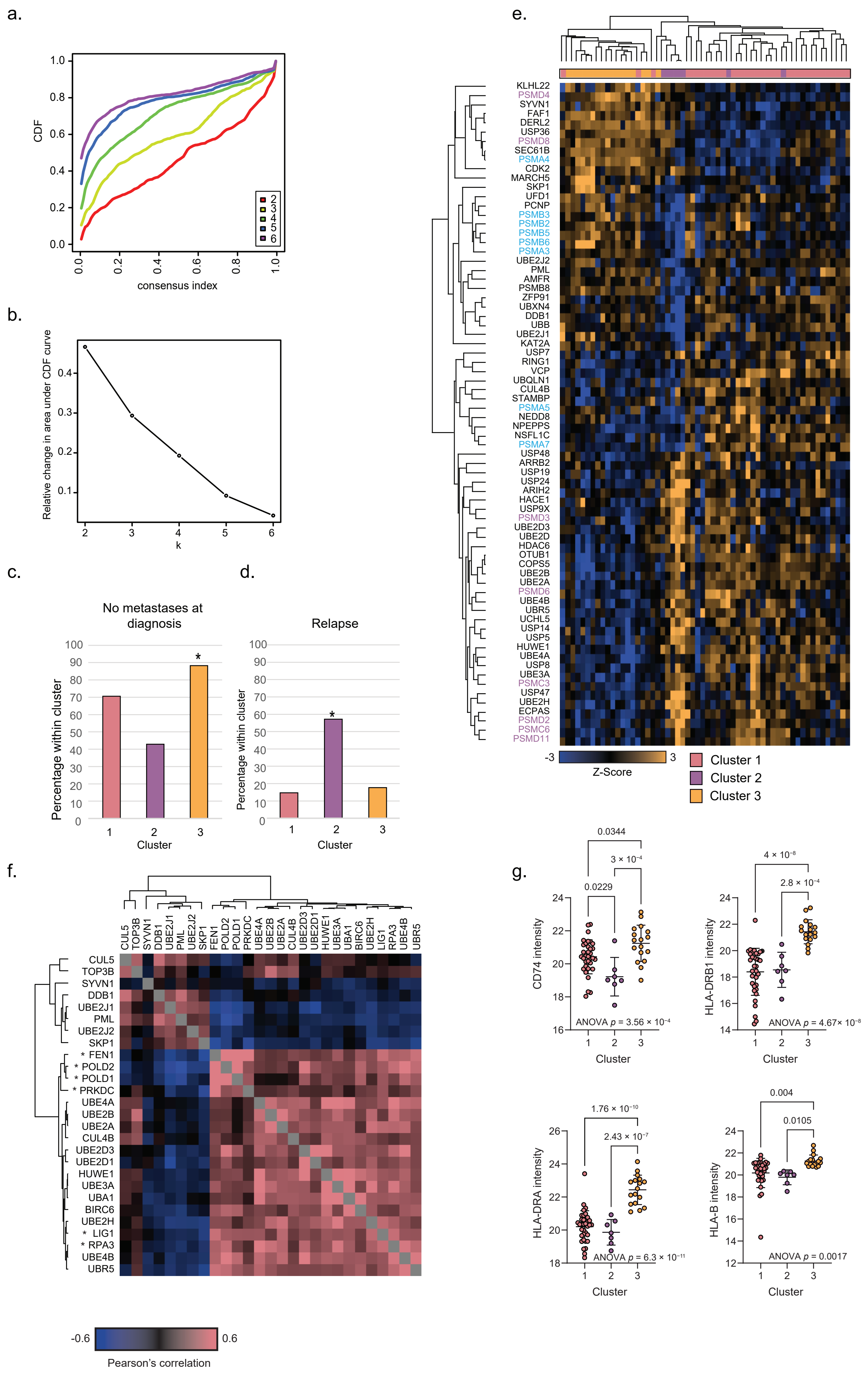

Supplementary Figure 5:

a) The cumulative distribution functions (CDF) of the consensus matrix for each k.

b) Relative change in area under CDF curve for k = 2–6.

c) Bar plot showing the percentage of patients diagnosed with non-metastatic disease in each cluster (*n* = 58 biologically independent samples; data were averaged for patients with multiple samples). Cluster 3 shows a significantly higher proportion of non-metastatic patients (two-sided Fisher exact test, $p = 0.0436$).

d) Bar plot showing the percentage of patients with relapsed tumors in each cluster. (*n* = 58 biologically independent samples; data were averaged for patients with multiple samples). Cluster 2 shows a significantly higher proportion of relapsed patients (two-sided Fisher exact test, $p = 0.025$.

e) Hierarchical clustering of proteins involved in proteasome and ubiquitin-mediated proteolysis (*n* = 58 biologically independent samples; data were averaged for patients with multiple samples). 20S proteins are in blue and 19S proteins are in purple. Heatmap colors represent standardized protein expression (Z-score).

f) Pearson correlation matrix between DNA-damage response proteins and ubiquitin-related proteins (*n* = 58 independent biologically independent samples; data were averaged for patients with multiple samples). Colors indicate the Pearson correlation coefficient (r), ranging from blue (negative correlation) to red (positive correlation).

g) Dot plots of MHC class II protein expression in EWS consensus clusters (*n* = total 58 biologically independent samples; data were averaged for patients with multiple samples: Cluster 1, *n* = 34; Cluster 2, *n* = 7; Cluster 3, *n* = 17). Statistical analysis was performed using one-way ANOVA to assess overall differences, followed by two-sided Tukey's post-hoc pairwise comparisons between consensus clusters. Data are presented as mean ± SEM.

Source data are provided as a Source Data file.

Gordon et al. Supplementary Figure 6

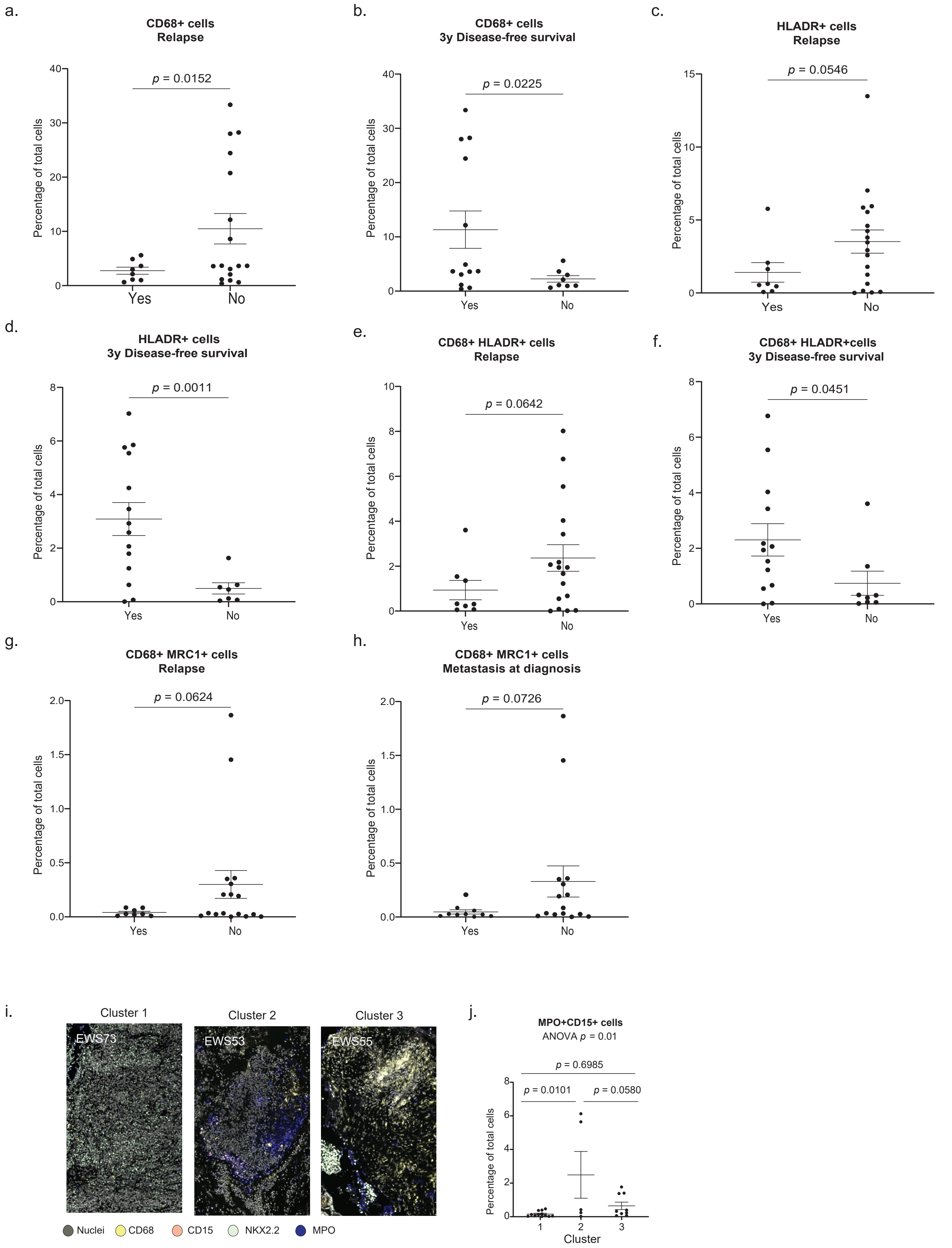

Supplementary Figure 6:

a-h) Analysis of CD68+, HLA-DR+, CD68+HLA-DR+, CD68+MRC1+ cells in biologically independent unique patient-derived samples with/without relapse (*n* = 8 relapse, 17 no relapse), 3y (3 years) DFS (n = 13 3y DFS, 8 no 3y DFS), or metastasis (n = 10 metastatic, 15 not metastatic). Data are presented as mean ± SEM. Two-sided Welch's t-tests were performed.

a,b) total CD68+ macrophages: relapse (*t* (17.68) = 2.6, *p* = 0.015, 95% CI [1.683, 13.81], *Glass' Δ* = 0.67), and 3y DFS (*t* (12.74) = 2.595, *p* = 0.015, 95% CI [-16.63, -1.503], *Glass' Δ* = 0.73).

c,d) HLA-DR^{+} cells: relapse (*t* (22.37) = 2.029, *p* = 0.054, 95% CI [-0.04514, 4.269], *Glass' Δ = 0.62*) and 3y DFS (*t* (15.73) = 3.987, *p* = 0.001, 95% CI [-3.968, -1.211], *Glass' Δ = 1.12*).

e,f) CD68+HLA-DR+ macrophages: relapse (*t* (22.80) = 1.945, *p* = 0.064, 95% CI [-0.09119, 2.946], *Glass' Δ*= 0.12) and 3y DFS (*t* (18.99) = 2.145, *p* = 0.045, 95% CI [-3.083, -0.03792], *Glass' Δ* = 0.74).

g,h) CD68^{+}MRC1^{+} macrophages: relapse (*t* (16.24) = 2, *p* = 0.062, 95% CI [-0.0151, 0.5337], *Glass' Δ* = 0.48) and metastasis at diagnosis (*t* (14.51) = 1.936, *p* = 0.072, 95% CI [-0.02940, 0.5946], *Glass' Δ* = 0.5).

i) Representative immunofluorescent images of myeloid cells stained for CD68, CD15, or MPO, and EWS cells stained with NKX2.2. Scale bar, 50 µm. Images are representative of n = 26 biologically independent patient-derived samples.

j) The proportion of CD15+MPO+ cells in three clusters was calculated as a percentage of the total cell population in the proteomic ROIs. Data are shown as mean ± SEM. Statistical analysis was performed using one-way ANOVA to assess overall differences (*F* (2, 23) = 5.259, *p* = 0.0132, η^2 = 0.31), followed by two-sided Tukey's post-hoc pairwise comparisons between clusters. P-values are indicated in the figure.

Source data are provided as a Source Data file.

Gordon et al. Supplementary Figure 7

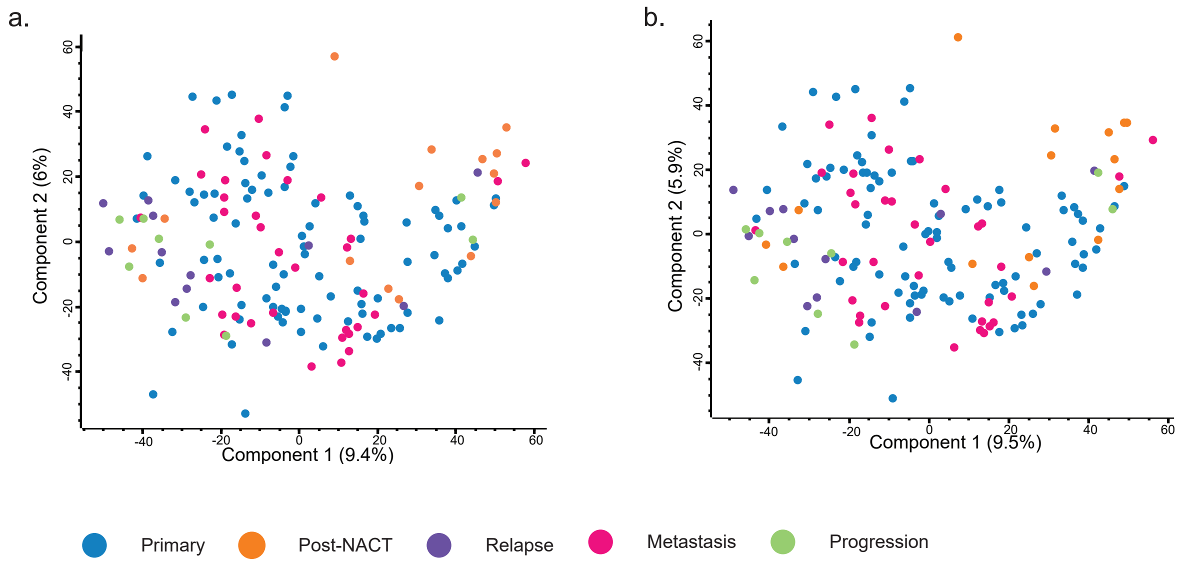

Supplementary Figure 7:

a) PCA analysis of the proteomic analysis of EWS cohort.

b) PCA analysis same as in (a) after removal of ECM proteins.
