# Supplementary material for: Proteomic landscape of Ewing sarcoma primary tumors and metastases
Source: Nat Commun. 2026 Mar 11;17:3802. doi: 10.1038/s41467-026-70449-5 (PMC13111610; doi:10.1038/s41467-026-70449-5)
Supplement: Supplementary file 3 — Description of Additional Supplementary Files [file 41467_2026_70449_MOESM3_ESM.pdf]

# Description of Additional Supplementary Files

**Supplementary Data 1:** EWS patients' clinical parameters.

**Supplementary Data 2:** All EWS proteomic runs annotated with clinical parameters.

**Supplementary Data 3:** Patients' clinical and pathological characteristics.

**Supplementary Data 4:** Processed proteomics data table. Protein intensities of different CVs were summarized and log2 transformed.

**Supplementary Data 5:** Primary samples with their clinical parameters for multiplexed immunofluorescence
