# Supplementary material for: Proteomic landscape of Ewing sarcoma primary tumors and metastases
Source: Nat Commun. 2026 Mar 11;17:3802. doi: 10.1038/s41467-026-70449-5 (PMC13111610; doi:10.1038/s41467-026-70449-5)
Supplement: Supplementary file 5 — Reporting Summary [file 41467_2026_70449_MOESM5_ESM.pdf]

nature portfolio

Corresponding author(s): Tamar Geiger

Last updated by author(s): Jan 21, 2026

Reporting Summary

Nature Portfolio wishes to improve the reproducibility of the work that we publish. This form provides structure for consistency and transparency in reporting. For further information on Nature Portfolio policies, see our Editorial Policies and the Editorial Policy Checklist.

Statistics

For all statistical analyses, confirm that the following items are present in the figure legend, table legend, main text, or Methods section.

n/a	Confirmed	
☐	☒	The exact sample size (*n*) for each experimental group/condition, given as a discrete number and unit of measurement
☐	☒	A statement on whether measurements were taken from distinct samples or whether the same sample was measured repeatedly
☐	☒	The statistical test(s) used AND whether they are one- or two-sided<br>*Only common tests should be described solely by name; describe more complex techniques in the Methods section.*
☐	☒	A description of all covariates tested
☐	☒	A description of any assumptions or corrections, such as tests of normality and adjustment for multiple comparisons
☐	☒	A full description of the statistical parameters including central tendency (e.g. means) or other basic estimates (e.g. regression coefficient) AND variation (e.g. standard deviation) or associated estimates of uncertainty (e.g. confidence intervals)
☐	☒	For null hypothesis testing, the test statistic (e.g. *F*, *t*, *r*) with confidence intervals, effect sizes, degrees of freedom and *P* value noted<br>*Give P values as exact values whenever suitable.*
☒	☐	For Bayesian analysis, information on the choice of priors and Markov chain Monte Carlo settings
☒	☐	For hierarchical and complex designs, identification of the appropriate level for tests and full reporting of outcomes
☐	☒	Estimates of effect sizes (e.g. Cohen's *d*, Pearson's *r*), indicating how they were calculated

Our web collection on statistics for biologists contains articles on many of the points above.

Software and code

Policy information about availability of computer code

Data collection	Proteomic data analysis was performed with DIA-NN version 1.8
Data analysis	Statistical analyses were performed in R, Prism or Perseus. The specific R packages and their versions are as follows: R version 4.3.2 with the packages survival: v3.8-3 (Used for Cox regression & Kaplan-Meier analysis), ConsensusClusterPlus: v1.66.0 (Used for Consensus clustering analysis), estimate: v1.0.13 (used for Tumor Purity/ESTIMATE score calculation), preprocessCore: v1.64.0 (Used for data normalization “Quantile”).<br>Image processing was performed with the following software tools: InFormV2.0.2, Akoya Biosciences (for spectral unmixing and background subtraction), Qupath v0.4.3 (for image stitching), Cellpose 2.0 (for nuclear segmentation), Qupath v0.5.1 (for fluorescent imaging classification and DAB staining quantification

For manuscripts utilizing custom algorithms or software that are central to the research but not yet described in published literature, software must be made available to editors and reviewers. We strongly encourage code deposition in a community repository (e.g. GitHub). See the Nature Portfolio guidelines for submitting code & software for further information.

Data

Policy information about availability of data

All manuscripts must include a data availability statement. This statement should provide the following information, where applicable:

- Accession codes, unique identifiers, or web links for publicly available datasets
- A description of any restrictions on data availability
- For clinical datasets or third party data, please ensure that the statement adheres to our policy

The raw mass spectrometry data generated in this study have been deposited in the PRIDE ProteomExchange database under accession code PXD050234 [https://www.ebi.ac.uk/pride/archive/projects/PXD050234]. The processed proteomics data generated in this study are provided in the Supplementary data. The Image data used in this study are available in the BioImage Archive database under accession code S-BIAD1597 [https://www.ebi.ac.uk/biostudies/bioimages/studies/S-BIAD1597?query=S-BIAD1597%20]. Source data are provided with this paper.

Research involving human participants, their data, or biological material

Policy information about studies with human participants or human data. See also policy information about sex, gender (identity/presentation), and sexual orientation and race, ethnicity and racism.

Reporting on sex and gender	Patient data includes the sex of the patients.
Reporting on race, ethnicity, or other socially relevant groupings	Such data is not available to us.
Population characteristics	All available data, which is approved by the ethics committees is reported in the supplementary material. Age data is provided as aggregated data to avoid patient identification.
Recruitment	Samples are collected from the pathology department. There is no active patient recruitment to the study.
Ethics oversight	The study was approved by the Institutional Review Board of Sourasky Medical Center (Approval No. 0122-11).

Note that full information on the approval of the study protocol must also be provided in the manuscript.

Field-specific reporting

Please select the one below that is the best fit for your research. If you are not sure, read the appropriate sections before making your selection.

☒ Life sciences ☐ Behavioural & social sciences ☐ Ecological, evolutionary & environmental sciences

For a reference copy of the document with all sections, see nature.com/documents/nr-reporting-summary-flat.pdf

Life sciences study design

All studies must disclose on these points even when the disclosure is negative.

Sample size	We used all available samples within a defined patient age.
Data exclusions	We excluded patients above 24 yo.
Replication	Biological replicates from the same patients are indicated in the supplementary Tables.
Randomization	Sample runs on the mass spectrometer were randomized.
Blinding	Data analyses were not blind.

Reporting for specific materials, systems and methods

We require information from authors about some types of materials, experimental systems and methods used in many studies. Here, indicate whether each material, system or method listed is relevant to your study. If you are not sure if a list item applies to your research, read the appropriate section before selecting a response.

Materials & experimental systems

n/a	Involved in the study
☐	☒ Antibodies
☐	☒ Eukaryotic cell lines
☒	☐ Palaeontology and archaeology
☒	☐ Animals and other organisms
☐	☒ Clinical data
☒	☐ Dual use research of concern
☒	☐ Plants

Methods

n/a	Involved in the study
☒	☐ ChIP-seq
☒	☐ Flow cytometry
☒	☐ MRI-based neuroimaging

Antibodies

Antibodies used

Three staining panels were used for immunofluorescence: i) Panel I with anti-MPO 480 (PA516672, Thermo Fisher Scientific, 1:100), anti-NKX2.2 (BSB3110, Bio SB, 1:75), anti-CD15 (MAS-11789, 481 Invitrogen, 1:50), anti-CD68 (CST-76437S, Cell Signaling Technology, 1:200); ii) Panel II with anti-NKX2.2 482 (as above), anti-CD4 (AB-ab133616-100, Abcam, 1:100), anti-CD8 (MA514548, Thermo Fisher Scientific, 483 1:100); iii) Panel 3 with anti-NKX2.2 (as above), anti-CD68 (as above), anti-HLAII (14-9956-82, Thermo 484 Fisher Scientific, 1:75), anti-MRC1 (HPA004114, Atlas Antibodies, 1:200), anti-PSMA3 (HPA000905, Atlas 485 Antibodies, 1:100). IHC analyses were performed with the following antibodies: anti-RRS1 (HPA060937, Atlas Antibodies, 488 1:1000), anti-NK2.2 (as above), anti-TFRC (136800, Thermo Fisher Scientific, 1:1200), anti-FTH1 489 (PA5257500, Thermo Fisher Scientific, 1:2000).

Validation

These antibodies validate mass spectrometric measurements that were performed on the same tissues.

Eukaryotic cell lines

Policy information about cell lines and Sex and Gender in Research

Cell line source(s)

A673 cells, RDES cells

Authentication

Cells were not authenticated.

Mycoplasma contamination

Tested negative.

Commonly misidentified lines
(See ICLAC register)

The cell lines used in this study are not listed as commonly misclassified cell lines according to ICLAC, Cellosaurus and other resources. A673 was originally misclassified as rhabdomyosarcoma and only later identified as EWS. As we use this line as an EWS model, the original misclassification does not impact this work.

Clinical data

Policy information about clinical studies
All manuscripts should comply with the ICMJE guidelines for publication of clinical research and a completed CONSORT checklist must be included with all submissions.

Clinical trial registration

NA

Study protocol

No clinical trial.

Data collection

Samples were collected from the Pathology Unit.

Outcomes

NA

Plants

Seed stocks

NA

Novel plant genotypes

NA

Authentication

NA
